# Polylactide-Based Films with the Addition of Poly(ethylene glycol) and Extract of Propolis—Physico-Chemical and Storage Properties

**DOI:** 10.3390/foods11101488

**Published:** 2022-05-20

**Authors:** Ewa Olewnik-Kruszkowska, Magdalena Gierszewska, Magdalena Wrona, Cristina Nerin, Sylwia Grabska-Zielińska

**Affiliations:** 1 Chair of Physical Chemistry and Physicochemistry of Polymers, Faculty of Chemistry, Nicolaus Copernicus University in Toruń, Gagarin 7 Street, 87-100 Toruń, Poland; mgd@umk.pl (M.G.); sylwiagrabska91@gmail.com (S.G.-Z.); 2 Department of Analytical Chemistry, Aragon Institute of Engineering Research I3A, University of Zaragoza, Torres Quevedo Building, María de Luna Street. 3, 50018 Zaragoza, Spain; magdalenka.wrona@gmail.com (M.W.); cnerin@unizar.es (C.N.)

**Keywords:** polylactide, propolis, food packaging, shelf life of products, blueberries

## Abstract

Polymeric films based on polylactide (PLA) with the addition of poly(ethylene glycol) (PEG) and a chloroformic extract of propolis were obtained. In the case of the studied films, polylactide (PLA) played the role of polymeric matrix and poly(ethylene glycol) was used as a plasticizer, while the extract of propolis was incorporated as a compound that could significantly affect the properties of the obtained materials, especially the water vapour permeation rate and the stability of the food products. Moreover, changes in structure, morphology, mechanical and storage properties as well as differences in colour, thickness and transparency after introducing propolis into the PLA–PEG system were determined. Based on the obtained results, it was established that the addition of the chloroformic extract of propolis significantly influences the most important properties taken into account during food packaging. It was also noticed that films with incorporated propolis were characterised by a significant improvement in the water vapour barrier property. Moreover, the obtained results prove that packaging containing a chloroformic propolis extract allow for the maintenance of the quality of the fruit stored for an extended period of time. To summarise, the application of a chloroformic propolis extract enables the formation of packaging materials that extend the shelf life of stored food products.

## 1. Introduction

Currently, most products need packaging, especially food. It can be clearly observed that polymers play a significant role in the packaging process. Active packaging deserves special attention among packaging materials; the purpose of active packaging is to prevent unfavourable changes in the quality and freshness of products. Active packaging includes antimicrobial packaging, containing various substances with properties that inhibit the growth of microorganisms. Active packaging also includes packaging with micro-perforation combined with a modified gas atmosphere.

One of the paths leading to obtaining active packaging materials is the incorporation of different compounds into a polymer matrix. With the aim of avoiding the accumulation of vast amounts of waste of packaging materials based on traditional plastics, biodegradable polymers have systematically been incorporated into the manufacturing process by the packaging industry. Currently, polylactides that can be obtained from renewable raw materials of plant origin are gaining popularity as packaging materials [1].

The most popular group of active substances are natural compounds of plant origin, such as essential oils (e.g., tea tree, cinnamon or clove essential oil) [2,3,4,5,6,7], bacteriocins (e.g., nisin) [8] or flavonoids (e.g., quercetin and catechin) [9,10,11,12]. Another active and natural compound that can be applied in packaging materials is propolis. Propolis is a waxy substance produced by honeybees during the pollen collection process from a variety of plants [13,14,15]. It has a characteristic fragrance and, depending on its plant origin, it can be orange, red, grey, dark green, brown or black. Depending on the storage temperature, propolis changes its state of aggregation: below 15 °C, it is hard and crumbles; in the range between 34.5 and 36 °C, it is soft and malleable; at approximately 70 °C, it becomes semi-liquid; and at approximately 90 °C, it melts. Bee putty has good solubility in ethyl alcohol (60–70%), acetone, ammonia, gasoline, ether and other organic solvents; however, it should be stressed that it does not dissolve in water [16,17,18]. Depending on the vegetation of the geographical area from which propolis originated, as well as the season and breed of the bees, its chemical composition varies. However, regardless of the source, the composition of propolis includes resins (40–50%), essential oils (14–16%), waxes (20–30%), pollen (5%), volatile substances (8–12%) and various pollutants [16,18]. The substances extracted from propolis belong to the so-called GRAS (generally recognized as safe). Bees collect propolis from Poland mainly from the black poplar (*Populus nigra*) leaf buds. About 65% of biologically active substances can be distinguished in it. In propolis, the most active substances are flavonoids (from 3.0 to 8.5%), as well as various phenolic and aromatic substances (about 77%) [16,17]. It is well known that propolis exhibits many pharmaceutical qualities, such as antibacterial, antifungal, antiviral and anti-inflammatory properties [13,14]. For this reason, interest in the use of propolis has been growing for many years [18].

In most cases, propolis extracts are introduced into polymeric solutions where films or coatings are formed as a result. In the article presented by Mascheroni [19], the diffusivity of propolis compounds in films obtained by the casting method of a solution containing ethyl acetate as a solvent, polylactide, polyethylene glycol 4000 and calcium bentonite was studied. The results indicated that the obtained materials can constitute possible delivery systems for food packaging applications. Zanella et al. [20] analysed fibres consisting of a poly(butylene-adipate-co-terephthalate)/poly(ε-caprolactone) blend with the addition of propolis. Electrospun fibre membranes based on polylactide and dichloromethane extracts of propolis were studied by Yan et al. [21]. The antibacterial effectiveness of the obtained electrospun membranes has been confirmed, particularly against *Staphylococcus aureus*. The bactericidal effects of propolis/polylactic acid nanofibres obtained via electrospinning were studied and published by Sutjarittangtham et al. [22]. The results of the bactericidal tests of the obtained nanofibres modified with propolis were highly satisfactory.

The characterization of composites comprising cellulose nanocrystals/poly(lactic acid) incorporated with *Tanacetum balsamita* essential oil and an ethanolic extract of propolis on the shelf-life extension of vacuum-packed cooked sausages was evaluated in the work of Khodayari et al. [23]. The authors proved that the films were capable of extending the shelf life of the cooked sausages by 50 days of refrigerated storage. The development of antimicrobial films based on poly(lactic acid) incorporating *Thymus vulgaris* essential oil and an ethanolic extract of Mediterranean propolis was conducted by Ardjoum et al. [24]. In this particular research, it was established that the combination of both propolis and essential oil leads to promising antimicrobial effects. In the work of Ucak et al. [25], microbiological, chemical quality and sensorial changes in rainbow trout fillets coated with gelatine films filled with an ethanolic extract of propolis were analysed. Based on the results, it was established that propolis extract can be recommended to be used as a natural antimicrobial additive.

Pullulan/chitosan-based edible composite films reinforced with zinc oxide nanoparticles and propolis used in meat packaging applications were developed by Roy et al. [26]. The presence of both compounds enhanced the water vapour barrier properties and antibacterial activity toward *E. coli* and *L. monocytogenes*.

In the case of propolis extract, mostly ethanol, ethyl acetate and ether/ethanol mixture are used as a solvent [19,23,24,25,27,28,29,30,31,32]. However, it has to be noted that the obtaining of PLA-based films from the mixture of solvents, such as chloroform and ethanol, can lead to the formation of an inhomogeneous structure [33]. For this reason, the aim of our work is to obtain polylactide-based films with the addition of poly(ethylene glycol) as a plasticizer and a propolis extract in one solvent, chloroform. The proposed method was described in our previous work, where the polylactide-based films with the addition of olive leaf extract in chloroform were studied [33]. In the present work, the structural (FTIR, AFM and SEM), mechanical, antibacterial and storage properties of polylactide-based films with the addition of PEG and propolis extract are studied. Moreover, the influence of propolis extract on the changes in colour, thickness, transparency and water vapour permeation rate are analysed. Taking into account that propolis contains several hundred different bactericidal substances, it is reasonable to assume that, as an additive to the packaging, it will positively influence the properties of the obtained materials and beneficially affect the extension of the shelf life of the products.

## 2. Materials and Methods

### 2.1. Materials

Polylactide, type 2002D with an average molecular weight of 79 kDa, was purchased from Nature Works^®^ (Minnetonka, MN, USA). Propolis was donated by the apiary of Father Eugeniusz Marciniak (Toruń, Poland). Poly(ethylene glycol) with an average molecular weight of 1500 g mol^−1^ was obtained from Sigma-Aldrich (Steinheim, Germany). Chloroform was purchased from Avantor Performance Materials Poland S.A. (Gliwice, Poland).

### 2.2. Obtaining Polylactide-Based Films

PLA-based materials were obtained using the casting and the solvent evaporation technique. First, 1.5 g of dry polylactide was dissolved in 50 mL of chloroform. Once the polylactide was dissolved, poly(ethylene glycol) (5% *w*/*w*, PLA) was added. Simultaneously, an extract of propolis was prepared in chloroform at a concentration of 14.2 mg/mL. Polylactide-based films containing propolis were obtained by introducing 5, 10 and 20 mL of propolis extract into the solution containing PLA and PEG. The resulting solutions were poured onto a Petri dish and evaporated at ambient temperature for 48 h. The composition of the obtained films is shown in Table 1.

### 2.3. FTIR Analysis

The chemical structure of the studied films was characterised by Fourier transform infrared spectroscopy (FT-IR). The spectra of individual samples were recorded in the frequency range between 650 and 4000 cm^–1^. Analyses were performed on a Nicolet iS10 spectrometer (Thermo Fisher Scientific, Waltham, MA, USA). The final spectra are the result of the application of a 4 cm^−1^ resolution and 64 scans for each sample. All spectra were analysed using OMNIC 7.0 software (Thermo Fisher Scientific, Waltham, MA, USA).

### 2.4. Scanning Electron Microscopy

Changes in the morphology of polymeric films caused by the addition of propolis were studied by means of a scanning electron microscope (Quanta 3D FEG, FEI Company, Hillsboro, OR, USA). Before the analysis, the samples were covered with a layer of gold. Micrographs of all samples were taken at 1000× and 5000× magnifications.

### 2.5. Atomic Force Microscopy

An AFM microscope with a scanning SPM probe of the NanoScope MultiMode type (Veeco Metrology, Inc., Santa Barbara, CA, USA) was used to obtain surface images of the obtained materials. Nanoscope software (Veeco Metrology, Inc. Santa Barbara, CA, USA) was applied to establish the roughness parameters, such as the root mean square (*R_q_*) and arithmetical mean deviation of the assessed profile (*R_a_*).

### 2.6. Mechanical Properties

The mechanical properties of the PLA-based films with and without the addition of propolis were analysed by means of the EZ-SX tensile tester (Shimadzu, Kyoto, Japan). The crosshead speed was 10 mm min^−1^. In the case of each type of the studied materials, at least five samples were studied. Based on the obtained results, the Young’s modulus (E), elongation at break (ε) and tensile strength (σ_m_) were established.

### 2.7. Thickness and Opacity

The thickness of the films was measured with an Absolute Digimatic Indicator, Sylvac S229 Swiss (Yverdon, Switzerland). The results are expressed as the average of twelve repetitions at different points of the studied samples.

The absorbance of the formed polymeric films with and without propolis at 550 nm (A_550_) was measured using a UV-Vis spectrophotometer (Ruili Analytical Instrument Company, Beijing, China). The absorbance at 550 nm was selected based on the method presented in the work of Suriyatem [34]. Absorbance measurements allowed to calculate opacity (*Op*) of the obtained materials according to Equation (1):

(1) $$\mathrm{Op}\;[{mm}^{-1}]=\frac{A_{550}}{d}$$

where *d* is the film thickness (mm).

### 2.8. Colour Change Analysis

The propolis extract can influence the changes in the colour of the obtained materials; for this reason, colour measurement was analysed using a MICRO-COLOR II LCM 6 (Dr Lange) colorimeter. To establish the colour difference (Δ*E*) of the obtained films, the CIE L*a*b* system was taken into consideration. It should be noted that *L* describes lightness and *a* represents parameter change from green to red, while *b* represents the colours ranging from blue to yellow. In an attempt to obtain reliable data, at least five measurements were performed for each of the samples; then, the average values were calculated. Parameters expressed as L*, a*, and b* belong to the control sample—in this case, an LG film.

The following Equations (2) and (3) were applied in order to estimate the yellowness index (*YI*) and total values of Δ*E*, respectively.

(2) $$YI=\frac{142.86\;\cdot \;b}{L}$$

(3) $$\Delta E=\sqrt{{\left(L-L^{*}\right)}^{2}+{\left(a-a^{*}\right)}^{2}+{\left(b-b^{*}\right)}^{2}}$$

### 2.9. Analysis of Water Vapour Transmission Rate and Water Vapour Permeation

The water vapour transmission rate (WVTR) of the obtained materials was determined according to the procedure developed by Grabska-Zielińska et al. [33]. The containers with CaCl_2_ covered with the LG film were used as control samples. The moisture penetration was established based on the changes in the CaCl_2_ weight measured at each 24 h for a total of 9 days. Before measurement, the films were conditioned by saturating with water vapour above NaCl saturated solution. The values of WVPR were calculated according to Equation (4), presented below:

(4) $$\mathrm{WVTR}=\frac{Slope\;of\;the\;straight\;line\;}{Surface\;area\;of\;the\;film}\;\left[\frac{g}{m^{2}\cdot h}\right]$$

Taking into account that the obtained films varied in thickness, the water vapour permeation was calculated using Equation (5):

(5) $$\mathrm{WVP}=\frac{WVTR\;x\;d}{\;∆Pv}\;\left[\frac{g\;mm}{m^{2}\cdot h\;kPa}\right]$$

where *d* is the film thickness (mm) and ∆*Pv* is partial pressure difference (3.218 kPa).

### 2.10. Storage of Blueberries—Weight Loss and Firmness

**Figure 1:**
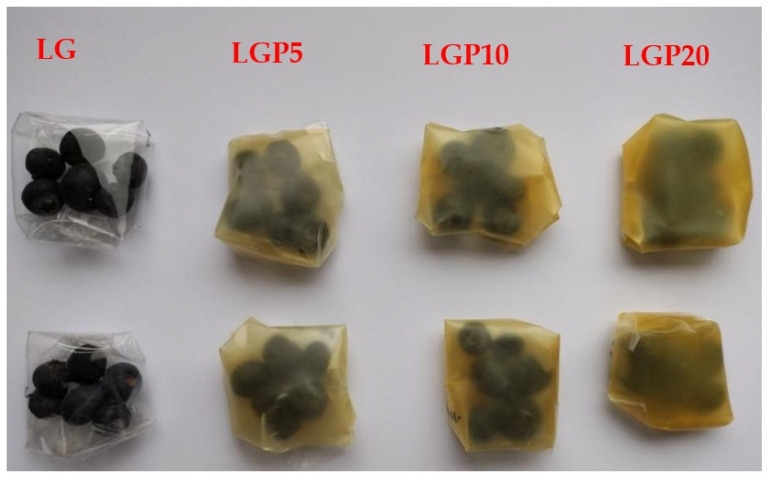
Blueberries packed into the obtained polymeric films.

Blueberries, originating from Chile, of the Biloxi variety free of any obvious physical damage were packed into envelopes made of the obtained polymeric films (Figure 1). Two envelopes were made from each material and 6 blueberries were packed in each of them. The formed packages were placed at 30 °C in a desiccator with a saturated NaCl solution (75% relative humidity) and stored for 14 days.

**Figure 2:**
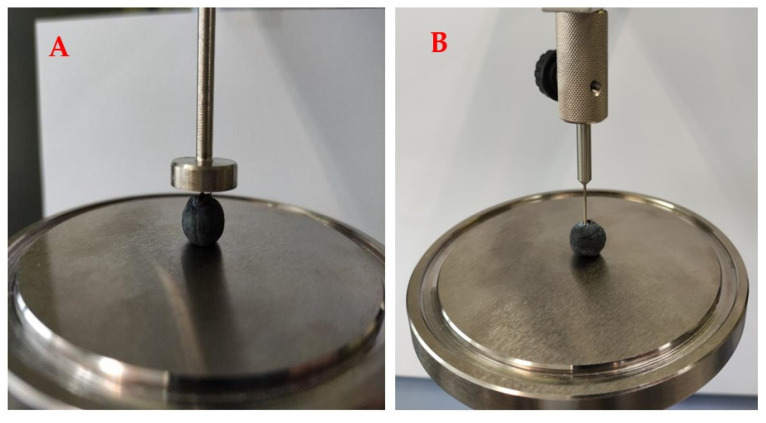
Equipment during (**A**) the compression test and (**B**) the piercing analysis of the blueberries.

With the aim of establishing weight changes, the samples were weighed after 4, 7, 11 and 14 days of storage. Moreover, the firmness of the blueberries before and after 14 days of storage was tested. For this reason, EZ-SX texture analyser (Shimadzu, Kyoto, Japan) was equipped with 50 N load cell, a cylindrical probe (diameter 20 mm) and a needle (diameter 2 mm) (Figure 2). During the compression test, the force required to obtain 20% compression of the fruits was registered. Furthermore, the force necessary to pierce the blueberries’ skin was established. The parameters of six blueberry fruits were measured in the case of the compression test as well as during the piercing analysis.

### 2.11. Statistical Analysis

Statistically significant differences between the properties of packaging without and with the addition of the propolis extract were assessed by a univariate analysis of variance (ANOVA) using GraphPad Prism 8.0.1.244 software (GraphPad Software, San Diego, CA, USA). Multiple comparisons between the means were performed with the statistical significance set at *p* ≤ 0.05.

## 3. Results and Discussion

### 3.1. FITR Analysis

**Figure 3:**
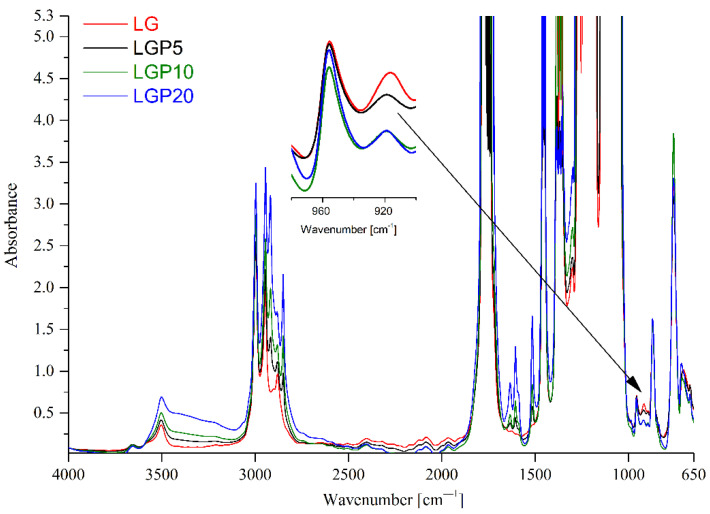
FTIR spectra sample with and without an addition of propolis extract.

Infrared spectroscopy is a very popular technique that uses the resonant absorption of electromagnetic radiation in the infrared range by vibrating groups of atoms present in the tested material. It is well known that individual groups of atoms absorb infrared radiation only with precisely defined photon energies. These energies depend on parameters such as mass, symmetry and the position of functional groups. The absorption spectrum obtained in the infrared range carries accurate information about the organic compounds present in the analysed sample. For this reason, FTIR technique was applied to establish the influence that the addition of propolis has on the structure of the formed polymeric films. In Figure 3, the FTIR spectra of all the obtained materials are depicted.

According to the literature [9,35], the following bands were observed for PLA and PEG structures: bands at 3659 cm^−1^ and 3501 cm^−1^ belonging to the -OH groups of the terminal units of polylactide and poly(ethylene glycol), respectively. The band at 2879 cm^−1^ corresponds to the vibration induced by the -CH_2_ group. The two bands at 2995 cm^−1^ and 2944 cm^−1^ are assigned to the asymmetric and symmetric stretching vibrations originating from the -CH_3_ group. The wide absorption band at 1757 cm^−1^ indicates stretching vibrations of the carbonyl group -C=O characteristic of ester groups, while the band at 1462–1446 cm^−1^ belongs to the asymmetric vibrations of the CH_3_ group. The two wide bands at 1276–1170 and 1140 cm^−1^ are assigned to a symmetrical—C–O–C—stretching vibration. The peaks at 871 cm^−1^ and 756 cm^−1^ correspond to C–COO stretching vibrations and CO deformation vibrations, respectively. Moreover, the bands that belong to the amorphous and crystalline phases at 915 cm^−1^ and 955 cm^−1^ can be observed [9].

The incorporation of propolis into the PLA––PEG system allowed new bands at 2917 cm^−1^ and 2849 cm^−1^ to be observed, which can be attributed to the vibration of the -C-H group. Moreover, additional, new vibrations were identified at 1634 cm^−1^, 1605 cm^−1^ and 1515 cm^−1^ that correspond to the stretching vibration of the -C=O group, C = C in the aromatics rings and the bending asymmetric vibrations of -N–H that are present in flavonoids and amino acids. The obtained results are in accordance with the works of Ulloa et al. [30] and Mot et al. [36], where propolis was applied as an active additive.

The chemical composition of polymeric films filled with propolis was also discussed in the work of Villalobos [37]. In this work, the addition of propolis nanoparticles caused a broad band to be registered from 3600 to 3000 cm^−1^, which was assigned to the -OH of phenolic compounds, such as cinnamic acid, caffeic acid and ferulic acid. The intensity of the described new bands increased with the propolis content in the obtained films [9,30]. Additionally, the spectra of the tested films after adding propolis show changes in the intensity of the bands corresponding to the amorphous and crystalline phases. The decrease in the band intensity at 915 cm^−1^ clearly indicates that propolis influences the crystallinity of the obtained materials.

### 3.2. Surface Morphology of the Studied Materials

Surface morphology and topography belong to the factor that can influence food adhesion [38]. For this reason, scanning electron microscopy and atomic force microscopy were used to analyse the morphology and surface topography of the PLA–PEG films with and without the addition of propolis extract.

**Figure 4:**
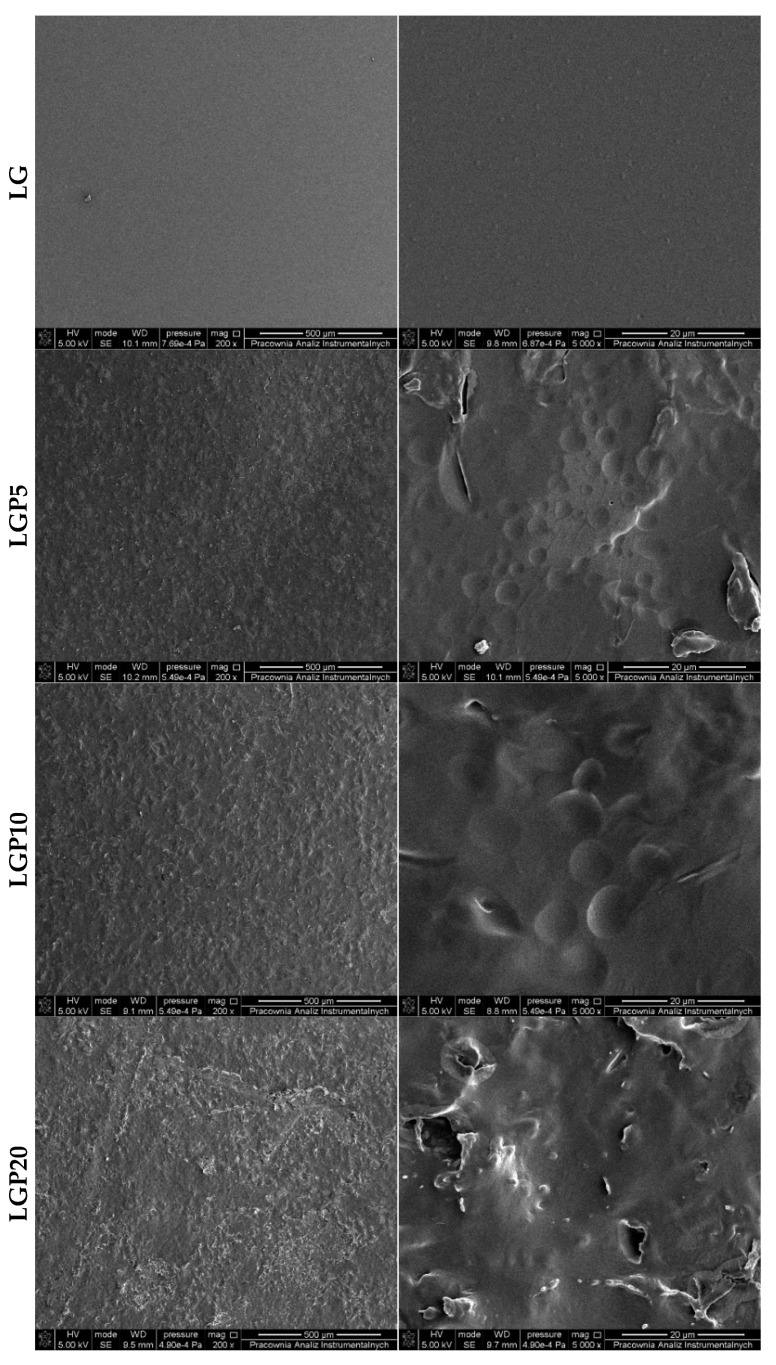
Surface images of the films with and without the addition of propolis extract.

The images of the LG film presented in Figure 4 show the limited miscibility of PLA and PEG, which is consistent with the results presented in the work of Richert et al. [35]. The introduction of propolis into the PLA–PEG matrix significantly influences the morphology of the obtained polymer films. The incorporation of 5 and 10 mL of propolis extract into the PLA–PEG system leads to the formation of holes and cavities. It seems very likely that they indicate the presence of volatile compounds in the composition of propolis.

The SEM images of the LGP20 sample (Figure 4) reveal that the surface of the studied material was covered with a significant number of depressions, rises and thickenings. In addition, along with an increase in the concentration of propolis in the films, an increase in the number of fissures, depressions, cracks and lumps on the surface was observed. A similar conclusion was reached by Villalobos [37], where scanning electron microscopy was applied to study the morphology of starch films infused with propolis nanoparticles. It was noticed that films containing propolis nanoparticles were characterised by a surface with a greater number of protrusions and elevations. Similar observations were also made by Suriyatem et al. [34], where SEM was performed for films consisting of rice starch and carboxymethyl chitosan with the addition of propolis extract. A rather heterogeneous structure with protrusions on the surface was noted.

**Figure 5:**
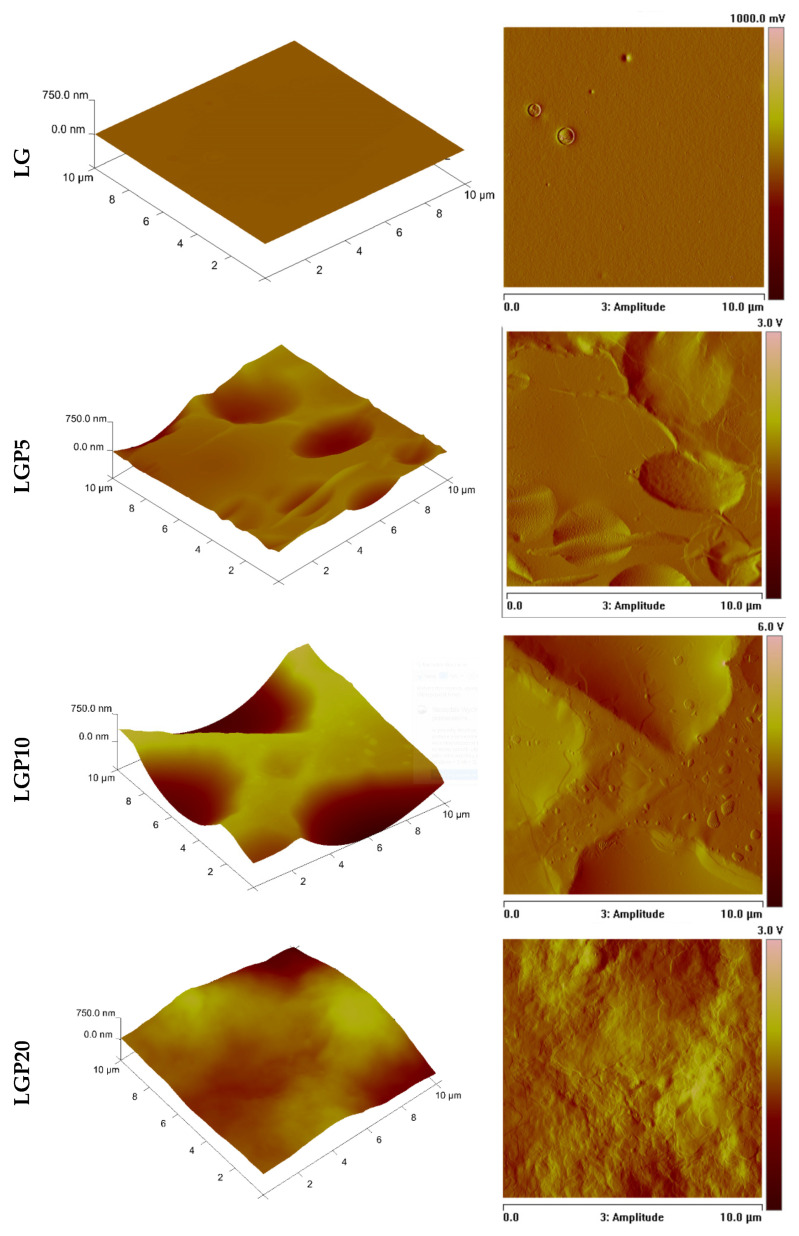
AFM pictures of the PLA–PEG-based materials with and without the addition of propolis extract.

The presence of propolis extract also significantly influences the roughness. For this reason, the surface topography was analysed by means of the AFM technique. In Figure 5, the three- and two-dimensional pictures of the surfaces of the obtained materials are depicted.

Moreover, the values of R_a_ (mean arithmetic deviation of the profile from the mean line), R_q_ (mean square deviation of surface roughness) and R_max_ (the maximum distance between the highest and lowest points of the recorded images) were calculated. The results presented in Table 2 clearly indicate that the addition of propolis extract significantly influences all studied roughness parameters. The addition of each amount of propolis extract leads to an increase in all the indicated parameters. The highest values of roughness parameters were achieved in the case of the LGP20 film. It should be noted, however, that the differences between R_a_, R_q_ and R_max_, established in relation to the film into which 20 mL of propolis extract was incorporated (LGP20), and the sample containing 10 mL of the active solution (LGP10) are negligible.

This finding is consistent with the results described in the work of Villalobos [37], where the increase in the roughness of starch-based films filled with nanoparticles was observed. Correa-Pacheco et al. [39] observed the heterogeneity of formed materials containing chitosan, chitosan nanoparticles, an ethanolic extract of propolis and glycerol; however, the roughness parameters in the case of the films incorporated with propolis decreased in comparison with the films made of chitosan and glycerol. This is important to consider, as the decreased roughness can be the result of an improved interaction between the components, most likely caused by the introduction of chitosan nanoparticles as well as the propolis extract. The obtained results suggest a clear correlation between the material’s composition and the roughness of the films’ surfaces.

### 3.3. Evaluation of the Mechanical Properties

**Figure 6:**
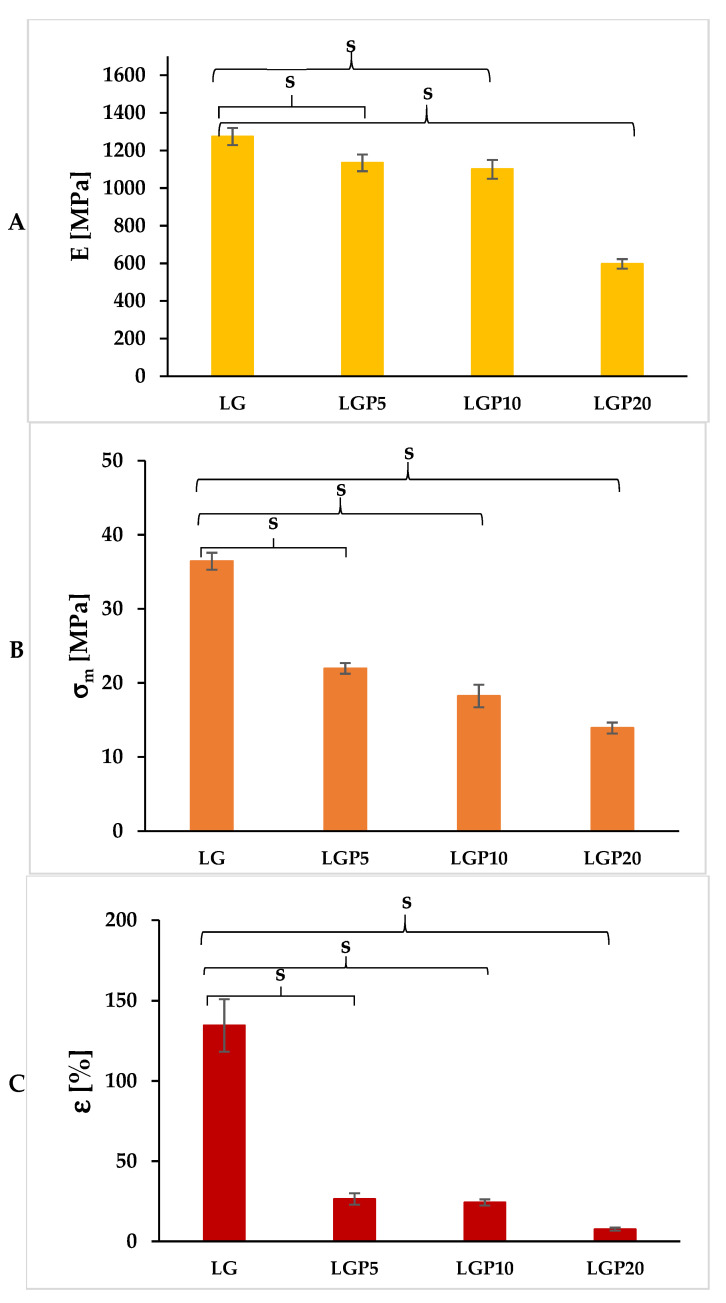
Mechanical properties of the studied polymeric films: (**A**) Young’s modulus (E), (**B**) tensile strength (σ_m_), (**C**) elongation at break (ε) (statistically significant differences *p* < 0.05 versus LG sample are indicated as s).

One of the most important functions of packaging is the protection of the contents. In the case of packaging films, elongation at break (ε), tensile strength (σ_m_) and Young’s modulus (E) count among the primary mechanical properties. The elongation at break reflects the film’s capacity to withstand mechanical strength without damage during production, handling or application. Tensile strength refers to the maximum stretch that the films can withstand, while Young’s modulus represents the stiffness of the film [40]. In Figure 6, the values of Young’s modulus, tensile strength and elongation at break are depicted. Based on the presented results, it was noted that the incorporation of propolis into the PLA–PEG system decreases the values of all of the studied mechanical properties.

The most significant decrease was observed in the case of elongation at break. It was found that the addition of as little as 5 mL of propolis extract leads to a reduction in the elongation at break value by about 80%. The lowest values of the parameters representing the mechanical properties were obtained in relation to the LGP20 sample. The Young’s modulus value for this material decreased by 54% in comparison to the LG film, while the tensile strength and elongation at break decreased by more than 62% and 94%, respectively, in relation to the film without propolis extract.

There are several potential reasons for the deterioration of the mechanical properties of the propolis-containing films. First of all, propolis can create strong intermolecular interactions with polymer chains, which disturbs their orientation while the film forms. Furthermore, the polyphenolic compounds contained in bee putty may form discontinuous areas in the film matrix, which reduces the film’s resistance to cracking [40,41].

The reduction in the Young’s modulus and in the tensile strength values was also observed in the work of Ulloa et al. [30]. However, the introduction of an ethanolic propolis extract, as well as powdered propolis into the polylactide matrix causes an increase in the elongation at break values. The same tendency was observed by Bodini et al. [31], where the properties of gelatine-based films with the addition of an ethanol–propolis extract were studied. Villalobos et al. [37] established that the introduction of propolis nanoparticles at a volume ranging between 0.5 and 1%, with respect to the starch mass, significantly improves the properties indicated above. The sample containing 3% of active compounds, however, was characterised by a remarkable decrease in the values of Young’s modulus, stress at break and deformation at break.

Based on the obtained results and as indicated in the literature, it was established that the amount of propolis is the key factor influencing the mechanical properties of the tested films. This limitation in the application of a higher concentration of propolis can be caused by the non-homogeneous distribution of additives as well as partial immiscibility.

### 3.4. Thickness and Opacity of the Obtained Films

Thickness and transparency, similar to the mechanical properties, belong to the most important features that are taken into account when choosing a packaging material. In Table 3, the thickness and opacity values of polymeric films with and without the addition of propolis extract are presented. Based on the obtained results, it was determined that the thickness of the obtained materials increases with the higher volume of propolis extract that is introduced into the PLA–PEG matrix. Taking into account that most of the additives lead to an increase in material thickness, the obtained results are not surprising [9,12,15].

The values of measured absorbance at 550 nm and the average thickness values allowed the calculation of the opacity of the obtained polymeric films. It can be clearly seen that both properties are correlated, with transparency decreasing in relation to the increasing amount of the propolis extract. It seems to be very likely that the reduction in transparency can be caused by the resins and waxes present in propolis [9,16]. The above-mentioned results indicate that opacity strongly depends on the composition of the analysed material. A similar tendency was observed in a publication focusing on materials containing an ethanolic propolis extract [30]. Taking into account that most food products are sensitive to light, the transparency decrease can be considered a positive effect. The obtained results justify a conclusion that films made of polylactide, poly(ethylene glycol) and a chloroformic propolis extract will prevent unfavourable phenomena occurring in food, such as discoloration, as well as nutrients and flavour losses.

### 3.5. Colour Parameters of the Studied Materials

The colour of food products, as well as that of packaging materials, are often taken into account by consumers. In most cases, the purchase of food is significantly connected with the product’s appearance. The introduction of different additives into the polymeric matrix often changes the visual parameters of potential packaging material. In the present work, the influence of the chloroformic propolis extract on the colour changes was evaluated. In Table 4, the calculated parameters, such as the yellowness index (*YI*) and total colour difference (Δ*E*), as well as *L*, *a* and *b* values, are presented.

The incorporation of propolis extract into the PLA–PEG system significantly affected all analysed parameters from the CIE LAB trichromatic colour model. Taking into account the *L*, *a* and *b* parameters, the highest differences were observed in the case the *b* values. It is well known that *b* values above zero indicate the yellowness of the sample, while values below zero correspond to the blue colour. It has been noted that, in the case of films containing propolis extract, the *b* parameter values are significantly higher than zero; therefore, all of the obtained films with an addition of propolis are visibly more yellow than the pure PLA–PEG film. Moreover, it should be highlighted that the *b* value increases with the increasing amount of propolis in the polymer matrix. The same tendency can be observed in the case of the yellowness index. The yellowing of the obtained films is due to the content of polyphenolic compounds present in propolis extract [34].

The polyphenolic substances influence the values of Δ*E*. As can be seen, the colour changes progress remarkably with the increase in the amount of propolis concentration in the PLA–PEG system. The highest Δ*E* value was observed in the case of the LGP20 sample where the film-forming solution contained 20 mL of the chloroformic propolis extract.

Similar observations were made in the publication of Roy et al. [26]. These data also correlate with the results described by Ardjoum et al. [24], where antimicrobial films based on poly(lactic acid) incorporated with Thymus vulgaris essential oil and an ethanolic extract of Mediterranean propolis were obtained. The same tendency was reported in the work of Ulloa et al. [30], where a theory that the quantity of propolis extract affects the values of the colour parameters was proved. Moreover, the observation mentioned above is in accordance with results described by Suriyatem et al. [34], where biodegradable rice starch/carboxymethyl chitosan materials containing propolis extract were studied.

### 3.6. Water Vapor Permeation Rate

**Figure 7:**
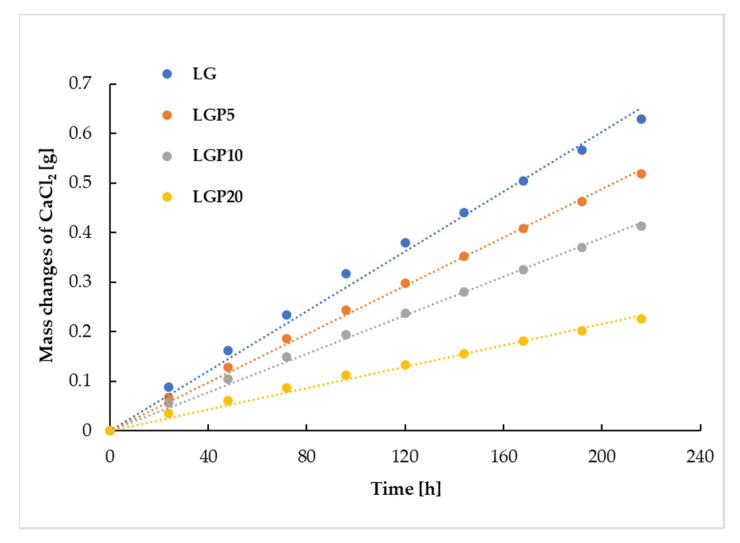
Changes in CaCl_2_ mass through time.

One of the factors that are taken into account when choosing components to be used in the formation of packaging materials is the resistance to water vapour permeability. Excessive water vapour penetration through the packaging film may cause the food to deteriorate and shorten the shelf life of the stored products. For this reason, in the present work, the influence of a chloroformic propolis extract on the water vapour permeation rate was investigated. With the aim of establishing the WVPR, the mass changes of stored CaCl_2_ were studied. In Figure 7, it can be clearly seen that, in all containers covered with the PLA–PEG-based films with and without the addition of propolis, the mass of CaCl_2_ increased linearly with the passage of time, during which the samples were stored in a container with a saturated sodium chloride solution.

It should be noted, however, that the most significant increase in the mass of calcium chloride occurred in the case of the water vapour penetration through the LG material, while the smallest increase was observed for the mass of CaCl_2_ covered with the LGP20 film.

**Figure 8:**
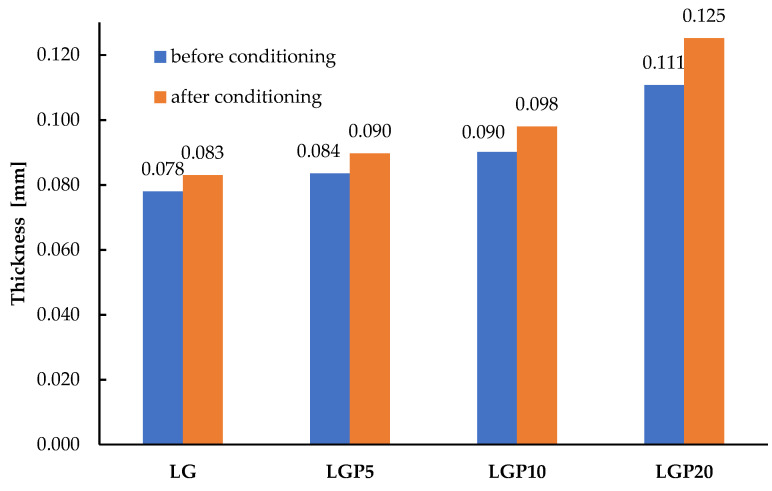
The thickness of the obtained polymeric films before and after conditioning.

As mentioned above, the films were conditioned to be characterised by a consistent thickness. The changes in thickness after storage in the desiccator under the controlled environmental conditions at 30 °C and 75% relative humidity were studied. It needs to be highlighted that, after the WVTR analysis, a change in the thickness of the materials was not noted. Based on the obtained results, it is reasonable to assume that changes in thickness were only caused by the sorption of water vapour in the structure of polymeric films during conditioning. The thickness changes can be seen in Figure 8.

**Figure 9:**
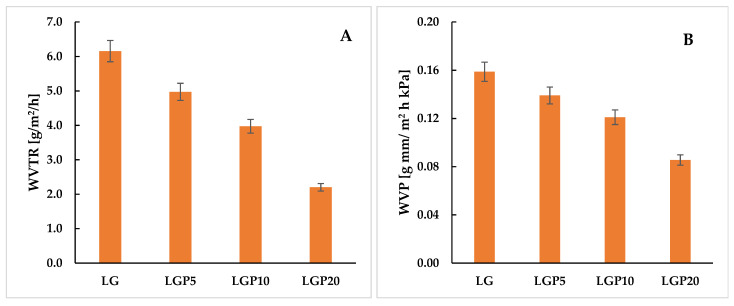
Influence of the propolis extract on the water vapour transmission rate (**A**) and water vapour permeation (**B**).

In Figure 9, the values of WVTR (A) and WVP (B) are shown. Based on the presented data, the influence of both the addition of propolis extract as well as the thickness of the formed films can be studied. It was noted that the introduction of the chloroformic extract of propolis significantly decreases the water vapour transmission rate. It was found that the addition of 20 mL of the prepared extract reduced the WVTR almost threefold. In the case of the LG sample, the WVTR value equals 6.15 (g m^−2^ h^−1^), while in relation to the LGP20 material, it was 2.20 (g m^−2^ h^−1^) only. According to the literature [40], two possible phenomena can be responsible for the significant decreases in the WVTR values. One of the factors is the reduction in the hygroscopicity of the obtained films. It is well known that propolis extract contains a large number of polyphenolic compounds as well as hydrophobic waxes, which can interact with the polymeric matrix. Moreover, it is reasonable to assume that propolis extract is capable of reducing the void space between the polymeric chains and thus limits the penetration of water vapour.

Taking into account that the introduction of the propolis extract caused an increase in the thickness of the obtained materials, this factor had to be considered during the analysis of the penetration passage of water vapour. For this reason, the water vapour permeation was calculated, and the results are shown in Figure 9B.

The results show that, despite the increase in the film thickness caused by the addition of the propolis extract, the obtained materials are still characterised by significantly lower WVP values in comparison with the LG film. The differences in the water vapour permeation value of the control film and the materials filled with propolis extract are not as impressive as in the case of WVTR; however, the WVP calculated for the LGP20 sample is still almost twice as low as in the case of the LG material.

This observation is in accordance with the results described in the work of Ulloa et al. [30]. The significant reduction in water vapour permeability after the introduction of propolis extract in comparison with the control film was observed in the work of Bodini et al. [31], where the properties of gelatine-based films with an added ethanolic propolis extract were studied.

Other researchers indicate that opposite scenarios are also possible. Moreno et al. [42] indicated that a decrease in the WVP of gelatine containing an ethanolic extract of propolis can be connected with the number and type of phenolic compounds. The same tendency was observed in the work of Suriyatem et al. [34], where the reduction in water barrier properties was explained as a result of the hydrophilic/hydrophobic ratio of the used compounds and the reduction in the density of the films. A similar effect was observed in the work of Hajinezhad et al. [43], where the barrier properties of packaging films based on low-density polyethylene with the addition of propolis were studied. The authors established that the rise in water vapour permeability can be the result of cracks and holes forming on the surface, which may, in turn, lead to the higher absorption of moisture.

To summarise, in our case, the addition of propolis extract significantly decreases the water vapour permeability of the PLA–PEG-based materials and allows for the safer and more efficient storage of food.

### 3.7. Blueberries’ Storage: Weight Loss and Firmness

It is well known that blueberries are characterised by having beneficial health properties. They improve memory, reduce the risk of hypertension, support sight and enhance digestive processes. Moreover, blueberries are also a remedy for cirrhosis of the liver and are even considered as a part of an anti-cancer prophylaxis. For this reason, they are generally perceived as a fruit that has a positive effect on the health of people. While shopping, in most cases, consumers have their own expectation for the texture of a particular product. This is also true in the case of various fruits. However, it is well known that an extended storage time leads to the development of fungi and the eventual decomposition of products. It is a commonly recognized fact that the type of packaging material and storage conditions are among the most important factors that impact the quality of food products [44].

**Figure 10:**
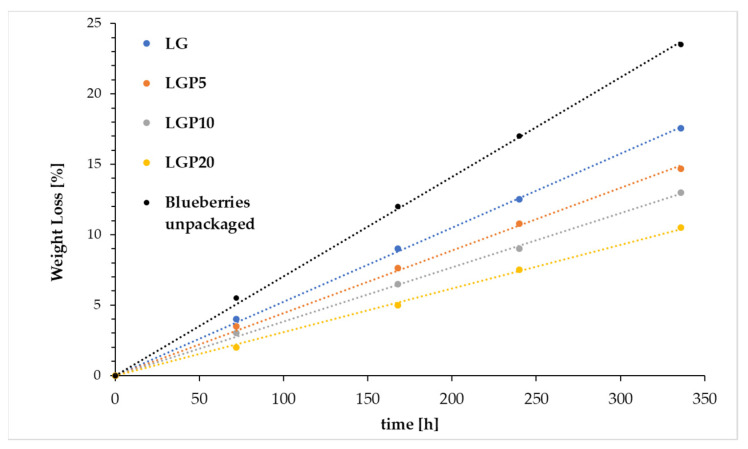
Weight loss of the unpacked and packed blueberries during storage.

With the aim of establishing the effects of storage time on the force required to squeeze the blueberries, a compression test under quasi-static loading and a skin piercing test were devised. Moreover, the blueberries’ weight loss during 14 days of storage was evaluated. In Figure 10, the weight loss of blueberries during storage time was shown. It can be clearly seen that an increased storage time causes the blueberries’ mass to decrease. Furthermore, it is worth noticing that, with an increase in the content of propolis extract in the PLA–PEG-based films, a decrease in the weight loss rate was observed. This phenomenon can be explained by the migration of water from the fruit to the environment [45]. The weight loss of blueberries packed in materials consisting only of polylactide and poly(ethylene glycol) reached about 20 wt.% after a storage period of 14 days, while the introduction of 20 mL of propolis extract in the PLA–PEG system allowed the weight loss to be limited to 15 wt.% Taking into account that the addition of propolis extract significantly increases the water barrier property, the obtained results are not at all surprising.

The same tendency was observed in the work of Jiang et al. [46], where the packaging materials made of chitosan and propolis extract were formed, and in the work of Pobiega et al. [47], where blueberries were coated with a polymeric film consisting of pullulan and propolis extract.

**Figure 11:**
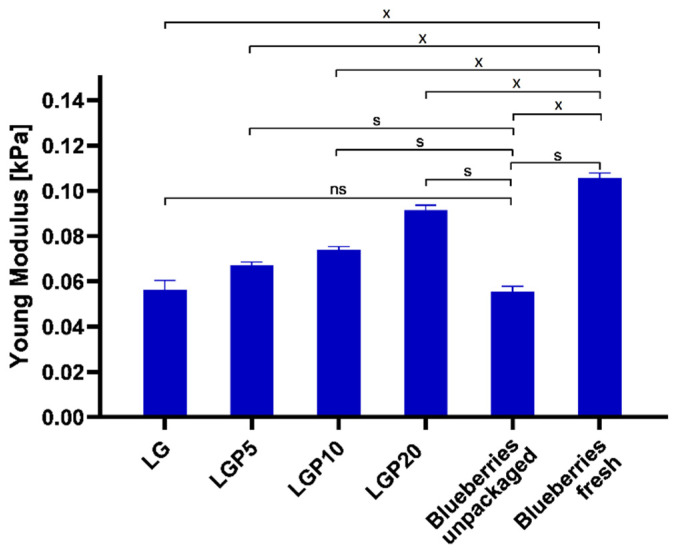
Compression test of the blueberries (*p* ≤ 0.05; ns—no statistically significant difference; s—statistically significant difference vs. blueberries unpackaged; x—statistically significant difference vs. blueberries fresh).

**Figure 12:**
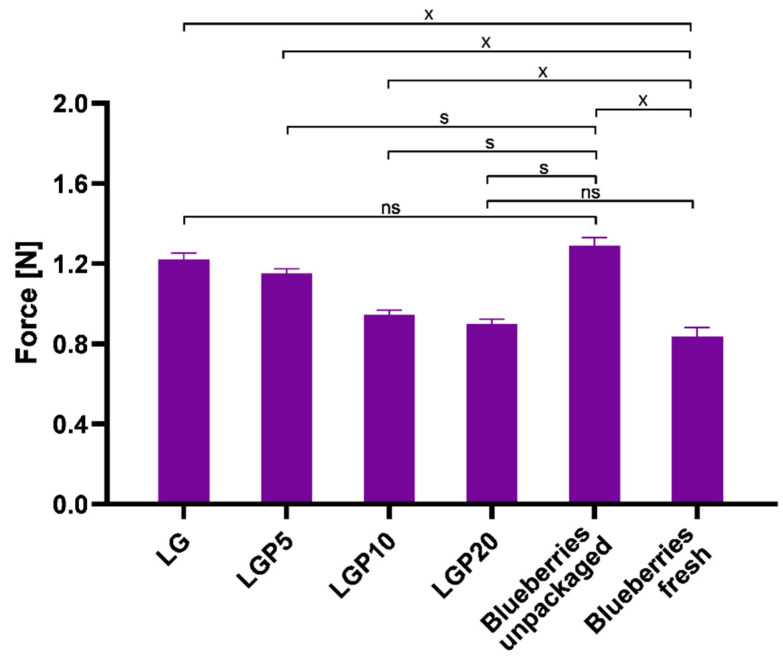
Puncture of blueberries (*p* ≤ 0.05; ns—no statistically significant difference; s—statistically significant difference vs. blueberries unpackaged; x—statistically significant difference vs. blueberries fresh).

Another factor that can indicate the changes in the quality of the fruit is the firmness. According to the definition presented in the work Ermiş [44], firmness is described as the maximum force that is necessary to obtain the assumed strain in compression or puncture. The changes in force values during the compression test as well as the skin piercing test are shown in Figure 11 and Figure 12, respectively. In the case of the compression test, the firmness was expressed as the force needed to achieve 20% deformation of a particular berry. Significant differences in the force required to compress packaged and unpackaged blueberries can be seen. Moreover, the increase in the content of propolis extract indicates that films containing a higher amount of propolis act in favour of the extension of the shelf life of the tested fruits.

A similar effect of propolis addition on the blueberries’ firmness during the piercing analysis was observed. It was proved that the introduction of a higher amount of propolis extract into the PLA–PEG matrix reduced the force required to puncture the skin of the blueberries.

This phenomenon is related to the less obstructed penetration of the needle probe through the relatively firm skin of the fruits. The lowest value of force required in order to penetrate the skin was obtained in the case of blueberries packaged into the LGP20 material. The obtained results suggest that the addition of propolis extract limits the softening of the packed fruit. Such properties were also observed in the work of Duan et al. [45], where the effect of different edible coatings on the firmness of blueberries was studied. The opposite effect was described by Abugoch et al. [48], where blueberries were coated in chitosan with quinoa protein and sunflower oil.

To summarise, the firmness changes of the analysed blueberries after storage in packaging materials with and without the addition of propolis extract significantly depend on the amount of the introduced additive. As the amount of propolis extract in the polymeric films increases, the applied force during the compression test also increases. Moreover, the obtained results reveal a dependence between the composition of the packaging materials and the force values during the puncture test. The films containing a higher amount of propolis extract caused the fruits to be more susceptible to piercing, which indicates a lower value of force, characteristic of a less malleable surface. The obtained results illustrate that the formed packaging containing propolis extract reduces the volatilization of water vapour and, in this way, significantly influences the firmness of the analysed fruits.

## 4. Conclusions

In the present work, polymeric films based on polylactide, poly(ethylene glycol) and chloroformic propolis extract were obtained. Based on the recorded results, it was established that propolis has a significant effect on the properties of the formed materials, extending the shelf life of the blueberries packed in the novel films.

It was proved that the introduction of the chloroformic propolis extract into the PLA–PEG system significantly increases the water vapour resistance. It is reasonable to assume that the presence of waxes in the composition of the propolis extract contributes to the observed properties. Moreover, it was determined that the changes in the colour, transparency and thickness of the obtained films strongly depend on the quantity of the incorporated propolis extract.

Furthermore, it was noted that the inclusion of propolis extract significantly affected the firmness of the packaged blueberries. It was established that the application of the materials containing propolis extract allowed it to maintain the firmness of the fruit for a longer time in comparison with the unpackaged blueberries as well as with fruit stored in the PG film.

It should be noticed, however, that the addition of propolis extract adversely affects the mechanical properties of the obtained materials. This phenomenon can be attributed to the low compatibility between the used polymers and propolis components.

## Figures and Tables

**Table 1:** Composition of investigated films (L—polylactide; G—PEG; P—propolis).

Sample	PLA [g]	PEG [g]	Propolis Extract [mL]
LG	1.5	0.075	0
LGP5	1.5	0.075	5
LGP10	1.5	0.075	10
LGP20	1.5	0.075	20

**Table 2:** Roughness parameters of the studied materials.

Sample	*R_q_* [nm]	*R_a_* [nm]	*R_max_* [nm]
LG	3.16 ± 0.79	2.45 ±0.62	71.10 ± 16.07
LGP5	98.87 ± 13.69	73.80 ± 10.40	649.33 ± 122.32
LGP10	262.00 ± 17.79	222.00 ± 14.84	1153.00 ± 141.14
LGP20	264.95 ± 63.96	227.63 ± 49.34	1024.25 ± 380.08

**Table 3:** Thickness and opacity values of the studied materials.

Sample	Thickness [mm]	*Op* [mm^−1^]
LG	0.0780 ± 0.0038	0.159 ± 0.07
LGP5	0.0841 ± 0.0050	0.162 ± 0.09
LGP10	0.0901 ± 0.0043	1.609 ± 0.08
LGP20	0.1107 ± 0.0046	1.745 ± 0.12

**Table 4:** Colour parameters (*L*, *a*, *b*, Δ*E* and *YI*) of the studied materials (statistically significant differences versus the LG sample were indicated as follows: ^S^ *p* < 0.05).

Sample	*L*	*a*	*b*	Δ*E*	*YI*
LG	92.5 ± 0.1	1.3 ± 0.1	−13.6 ± 0.1	-	−21.0
LGP5	88.5 ± 0.3 ^S^	−2.6 ± 0.1 ^S^	7.5 ± 0.2 ^S^	21.8 ± 0.4	12.1
LGP10	86.7 ± 0.4 ^S^	−2.1 ± 0.1 ^S^	13.3 ± 0.2 ^S^	27.7 ± 0.3	21.8
LGP20	86.1 ± 0.2 ^S^	−3.6 ± 0.1 ^S^	18.8 ± 0.2 ^S^	33.4 ± 0.2	31.2

## Data Availability

Data is contained within the article.
